# Investigating How the Properties of Electrospun Poly(lactic acid) Fibres Loaded with the Essential Oil Limonene Evolve over Time under Different Storage Conditions

**DOI:** 10.3390/polym16071005

**Published:** 2024-04-07

**Authors:** Leah Williams, Fiona L. Hatton, Maria Cristina Righetti, Elisa Mele

**Affiliations:** 1 Department of Materials, Loughborough University, Loughborough LE11 3TU, UK; f.hatton@lboro.ac.uk; 2 National Research Council-Institute for Chemical and Physical Processes (CNR-IPCF), Via Moruzzi 1, 56124 Pisa, Italy; cristina.righetti@pi.ipcf.cnr.it

**Keywords:** electrospinning, polymer ageing, essential oil, limonene, poly(lactic acid)

## Abstract

Essential oils have been identified as effective natural compounds to prevent bacterial infections and thus are widely proposed as bioactive agents for biomedical applications. Across the literature, various essential oils have been incorporated into electrospun fibres to produce materials with, among others, antibacterial, anti-inflammatory and antioxidant activity. However, limited research has been conducted so far on the effect of these chemical products on the physical characteristics of the resulting composite fibres for extended periods of time. Within this work, electrospun fibres of poly(lactic acid) (PLA) were loaded with the essential oil limonene, and the impact of storage conditions and duration (up to 12 weeks) on the thermal degradation, glass transition temperature and mechanical response of the fibrous mats were investigated. It was found that the concentration of the encapsulated limonene changed over time and thus the properties of the PLA–limonene fibres evolved, particularly in the first two weeks of storage (independently from storage conditions). The amount of limonene retained within the fibres, even 4 weeks after fibre generation, was effective to successfully inhibit the growth of model microorganisms *Escherichia coli*, *Staphylococcus aureus* and *Bacillus subtilis*. The results of this work demonstrate the importance of evaluating physical properties during the ageing of electrospun fibres encapsulating essential oils, in order to predict performance modification when the composite fibres are used as constituents of medical devices.

## 1. Introduction

Essential oils (EOs) are complex mixtures of volatile compounds which are typically extracted from the non-woody parts of plants. They have attracted interest for use as bioactive agents in a range of industries due to possessing anti-bacterial, anti-fungal, anti-oxidation and anti-inflammation properties [1,2,3,4,5,6,7,8,9,10,11]. EOs are used as antimicrobial agents, as their wide natural availability and low degree of toxicity overcome the limitations associated with synthetic antimicrobials [1,9]. In recent years, the concept of encapsulating EOs within polymeric nanofibres produced by electrospinning has gained momentum, due to the possibility of achieving a longer and more controlled release of bioactive agents compared to other delivery methods [8].

Electrospinning is the process by which a dry network of polymer nanofibres are obtained from an electrically charged polymer solution as it is electrostatically attracted to a collector plate [12,13]. It has received growing interest for a number of reasons, including its compatibility with a wide range of natural and synthetic biopolymers and its ability to produce nanofibres with engineered properties to target specific applications [12,13,14,15,16]. In particular, electrospun nanofibres are considered to be applicable as innovative wound dressings as they physically protect the wound bed, absorb wound exudates and mimic the native structure of the extracellular matrix (ECM), all while supporting cell proliferation [9,10,12].

It has been demonstrated that electrospun mats containing phytotherapeutic agents like EOs are promising as advanced wound dressings due to the inclusion of additional functionality, such as displaying antibacterial characteristics. Looking to the literature, fibrous mats containing cinnamon [1,2], lemongrass [1], peppermint [1], tea tree [3], manuka [3,4], thyme [5], lavender [6], chamomile [7], limonene [9], clary sage oil and black pepper oil [9,10], and tangerine peel [11], to name a few examples, have been generated and the properties of the resulting EO-loaded nanofibre mats investigated. The key findings reiterated throughout these studies have established that EOs impart bioactivity to electrospun fibres by making them, for example, effective for the prevention of bacterial colonisation and skin inflammations, and for the inhibition of oxidative processes [8]. Also, the inclusion of EO within the fibres has been linked with generating varying surface topographies thought to arise from thermodynamic instabilities and resulting phase separation phenomena [9,10]. Some of the textures reported include nanopores, wrinkled surfaces and nanofeatures when limonene, clary sage oil and black pepper oil were used, respectively. Textured surfaces are known to enhance cell adhesion and proliferation, and as such, dressings containing EOs should be well suited to accelerating the tissue repair mechanisms and thus also prevent a wound from becoming chronic [1,3,4,5,9].

As reported in the literature, limonene can be effectively encapsulated into the electrospun nanofibres of poly(vinyl alcohol) (PVA) [17,18,19], fish gelatin [20] and PLA [9]. The use of water-soluble polymers like PVA required an emulsification process to form limonene droplets within PVA-water solutions before electrospinning (due to phase separation between water and limonene). When PLA was the polymeric matrix, instead, this extra processing step was not needed because the use of organic solvents, like acetone, chloroform or DMF, promoted the formation of PLA–limonene solutions rather than emulsions. 

Despite the promising findings cited in the literature, the effects of EOs on the properties of the electrospun fibres over time periods longer than 14 days have not yet been investigated. Studies of this nature are necessary to develop systems which maintain their functionality (through maintaining EO concentrations in the fibres whilst not degrading the polymer) long enough to have clinical relevance. EOs are known to be volatile under ambient conditions, and so evaporate from the fibres over time. This is characterised in the literature for shorter time periods (180 min) [2,5,6,7] up to a maximum of 14 days [4]. Across all studies, the rate of release of EO was seen to decrease over time, with the greatest release occurring immediately after fibre synthesis. EO release has been seen even at the end of a 14-day test duration [4], suggesting that the effect of topical EO application can be maintained for at least as long. This was demonstrated using a rabbit model, and the beneficial effects of EO-loaded fibres in promoting wound healing were seen with an accelerated healing rate compared to wounds not treated with EOs, over a duration of 14 days [5]. Quantifying the concentration of EO retained within electrospun fibres for longer durations allows for predictions to be made about treatment efficacy over time. Furthermore, as a certain concentration of EO is needed within the fibres in order for treatments to be effective, investigations into how to store the fibres to increase the retention of EOs over time are also clinically relevant. Here, these aspects together with changes in fibre characteristics (thermal properties and mechanical behaviour) are investigated, for the first time, over a period of 12 weeks. 

The EO selected in this work is limonene, which is found in the peel of citrus fruits, and is primarily used in the food industry as a flavouring or scent, in the cosmetic industry to facilitate cream permeation into the skin, and in medical applications for its, among others, anti-cancer properties [21]. Electrospun systems containing limonene have been shown to have good antimicrobial activity against species like *Staphylococcus aureus*, *Staphylococcus epidermis*, *Escherichia coli* and *Pseudomonas aeruginosa* [9,10,22]; antifungal properties have also been noted [23]. These properties have raised interest in using limonene clinically to inhibit the proliferation of species that are responsible for infections in hospital settings, such as *P. aeruginosa* and *E. coli* [24].

In order for a system to be suitable for clinical use, it must have a sufficiently long shelf life to allow for convenient transport and storage, whilst simultaneously maintaining product quality. In this study, the role of limonene on the stability of poly(lactic acid) (PLA) electrospun nanofibres was investigated over time, particularly focusing on the changes in thermal and mechanical properties, as well as antibacterial activity. The impact of storage conditions and duration on the performance of limonene-loaded PLA fibres was investigated, for the first time, over a period of 12 weeks. Also, the suitability of the composite electrospun mats as antibacterial material was assessed using the model microorganisms *Escherichia coli*, *Staphylococcus aureus* and *Bacillus subtilis*. Through conducting such investigations, a knowledge gap is addressed, with the aim of facilitating the transition to clinical uses of these promising limonene-functionalised electrospun nanofibrous systems.

## 2. Materials and Methods

### 2.1. Materials

Poly(lactic acid) (PLA 4060D, MW = 120,000 g/mol, amorphous polymer with an L-lactide content of around 88 wt%) was purchased from NatureWorks LLC (Minnetonka, MN, USA); (R)-(+)-Limonene (C_10_H_16_ MW = 136.23 g/mol) and acetone was purchased from Sigma-Aldrich (Gillingham, UK). All chemicals were used without further purification. 

LB Broth (MILLER) (Sigma-Aldrich^TM^ Solutions, Merck KGaA, Darmstadt, Germany) was purchased in powder format and was mixed as per the manufacturer’s instructions to make liquid LB broth culture media. Ringer’s solution was prepared as per the manufacturer’s instructions by dissolving RINGER tablets (Millipore^TM^ Solutions, Merck KGaA, Darmstadt, Germany) in deionised (DI) water. Bacteriological Agar (powder form, Sigma-Aldrich^TM^ Solutions, Merck KGaA, Darmstadt, Germany) was added at a 1.5 *w*/*v*% ratio to LB broth to generate a firm-set solid agar substrate to culture the respective microorganisms on. *Escherichia coli* (ATCC 25922), *Staphylococcus aureus* (ATCC 19685) and *Bacillus subtilis* (ATCC 6633) were cultured as per the manufacturer’s instructions; cryopreserved bacteria frozen at −20 °C in 50% glycerol were used to generate the starter cultures described within this work.

### 2.2. Electrospinning Process

Solutions for electrospinning were prepared by dissolving PLA in acetone at a concentration of 14 *w*/*v*% for a minimum of 20 h. This polymer solution was used to produce PLA fibres (no limonene). To generate PLA nanofibres loaded with the essential oil limonene (PLA–Lim), limonene was added to the PLA solution (prior to electrospinning) at a concentration of 10 *v*/*v*% [9].

For the electrospinning process, a 5 mL syringe with a 21G needle was filled with the prepared polymer solution (either PLA/acetone or PLA/acetone/limonene) and was connected to a syringe pump (PHD ULTRA, Harvard Apparatus, Holliston, USA). The needle was clamped to the positive electrode of a high voltage power supply (S1500032-0, Linari Engineering s.r.l., Pisa, Italy) and the ground electrode was connected to an aluminium collector plate. Through observation by scanning electron microscopy (SEM, JSM-7800F, JEOL, Tokyo, Japan), it was determined that the optimal parameters for producing bead-free polymer nanofibres were a flow rate of 0.7 mL/h, a voltage of 12 kV and a needle tip to collector plate distance of 14 cm. Electrospinning was conducted for a time of 25 min; this allowed for a sufficient quantity of nanofibres to be collected. All experiments were carried out in normal, ambient laboratory environmental conditions (~20 °C).

### 2.3. Definition of Fibre Groups and Outline of Testing Timelines

Electrospun fibres were produced with and without the addition of limonene and they were tested at different timepoints between the time of generation and 12 weeks, as indicated in Table 1. To take into account possible changes to the properties of the electrospun mats, different storage conditions were investigated. Most electrospun samples were stored in an open system (normal conditions) achieved by placing them in closed Petri dishes (but not sealed). A portion of the PLA–Lim mats were stored in closed systems (sealed conditions) by wrapping the specimens in aluminium foil and placing them within Petri dishes that were subsequently closed from the environment by using parafilm. Also, when preparing samples for analysis by differential scanning calorimetry (DSC), fibres were sealed in DSC pans immediately after electrospinning (time 0), which also acted as a system closed to the environment. Details of the different groups of samples prepared, their reference names and the timepoints at which data were collected for each group of fibres are found in Table 1.

### 2.4. Characterisation of the Electrospun Fibres

The electrospun fibres were prepared for SEM observation by mounting the prepared specimens on adhesive carbon tabs and applying a coating of Au/Pd (20:80 ratio) under a 90 s run profile. Images were taken using a Field Emission Scanning Electron Microscope (FE-SEM) (JSM-7800F, JEOL) with a 2 kV beam. 

Thermogravimetric analysis (TGA) tests were carried out using a Discovery TGA 55 TA Instruments thermal analyser (New Castle, DE, USA). Samples weighing around 5–10 mg were heated under a dynamic mode. Measurements were performed at a heating rate of 10 °C·min^−1^ from room temperature (20 °C) to 500 °C under a nitrogen atmosphere in order to prevent any thermoxidative degradation. The weight loss of samples was measured as a function of temperature. TGA measurements were used to determine the concentration of limonene contained within the fibres after electrospinning and storage.

Thermal analysis was conducted by performing Differential Scanning Calorimetry (DSC) using TA Instruments Q200 Differential Scanning Calorimeter (New Castle, DE, USA) in a nitrogen atmosphere. Each type of mat was sealed in an aluminium pan and heated from −40 °C to 80 °C at a rate of 10 °C min^−1^. The glass transition temperature (*T*_g_) was taken as the midpoint of the heat flow rate increment associated with the glass-to-rubber transition. For each sample, a minimum of three repeat measurements were performed and an average taken. As detailed above, the specimens due to be tested under normal conditions were left to age before being placed in the sealed DSC pans at the time of testing. Specimens due to be tested under sealed conditions were prepared at a time equal to 0 weeks. The glass transition temperature of the PLA nanofibres (with and without limonene) was measured at each timepoint of testing. 

For analysis of the mechanical properties, the fibrous mats were detached from the aluminium foil and were cut into 0.5 cm × 4.0 cm strips. At each timepoint of testing, 15 strips from each batch of nanofibres were tested under tension until failure. Tensile tests were carried out using a single column tabletop Instron system (Instron 5944 fitted with a 2 kN loadcell) at room temperature (around 20 °C). A gauge length of 2.0 cm and an extension rate of 12 mm/min were used to perform the testing. Side action grip clamps with flat jaw faces were used to secure the specimens in place for testing.

### 2.5. Antibacterial Investigations 

The effect of limonene on bacterial growth inhibition was investigated with the following bacteria that can cause complications in wound care: *E. coli* (Gram negative), *S. aureus* (Gram positive) and *B. subtilis* (Gram positive). The AATCC test method 100–2004 (viability loss) was used to quantitatively assess the antibacterial properties of the electrospun mats [25]. First, LB broth (in a ratio of 2.5 g of LB broth powder per every 100 mL of DI water), molten LB agar (in a ratio of 2.5 g of LB broth powder and 1.5 *w*/*v*% bacteriological agar per every 100 mL of DI water) and Ringer’s solution (in a ratio of 1 tablet per 500 mL of DI water) were prepared and sterilised in an autoclave for 15 min at 121 °C. After the temperature of the LB broth had cooled to room temperature, the bacterial cultures (one with each of the different test strains being tested) were established by inoculating 100 mL of sterile LB broth with 1 mL of a starter culture of the target bacteria. The bacterial cultures were left to grow overnight at 37 °C in a rotary shaker at 150 rpm (CERTOMAT^R^ BS-1, SartoriusStedim Bioctech, Surrey, UK). Agar plates were prepared by pouring molten LB agar into Petri dishes and leaving it to set at room temperature. The overnight bacterial cultures were diluted by a factor of six by sterilised Ringer’s solution. A volume of 1.5 mL of each bacterial dilution was put into a glass vial, each already containing 20 mg of UV-sterilised electrospun PLA nanofibres (either with or without limonene) cut into small pieces at the bottom of the vial. The glass vials were thoroughly shaken for 10 s. Following this initial exposure to the material, 30 μL of bacterial dilution was taken from each vial, spread onto the freshly prepared agar plates and incubated at 30 °C for 24 h (“0 h contact time” samples). After incubating the bacterial dilution at 30 °C for 24 h, another 30 μL of the bacterial dilution was taken from each vial, spread onto agar plates and incubated for 24 h at 30 °C (“24 h contact time” samples). The colony-forming units (CFU) were counted for “0 h contact time” and “24 h contact time” samples, and from this the bacterial loss in viability was calculated using the formula:

$$Loss\;of\;viability\;\left(\%\right)\;=\;\frac{\left(B-A\right)}{B}\times 100\%$$

where *A* is the number of CFU counted for “24 h contact time” samples, and *B* is the number of CFU counted for “0 h contact time” samples. Each test was run in triplicate. The test was repeated every week to collect data from time = 0 weeks until time = 4 weeks, using PLA–limonene samples stored inside unsealed containers. 

## 3. Results and Discussion

**Figure 1:**
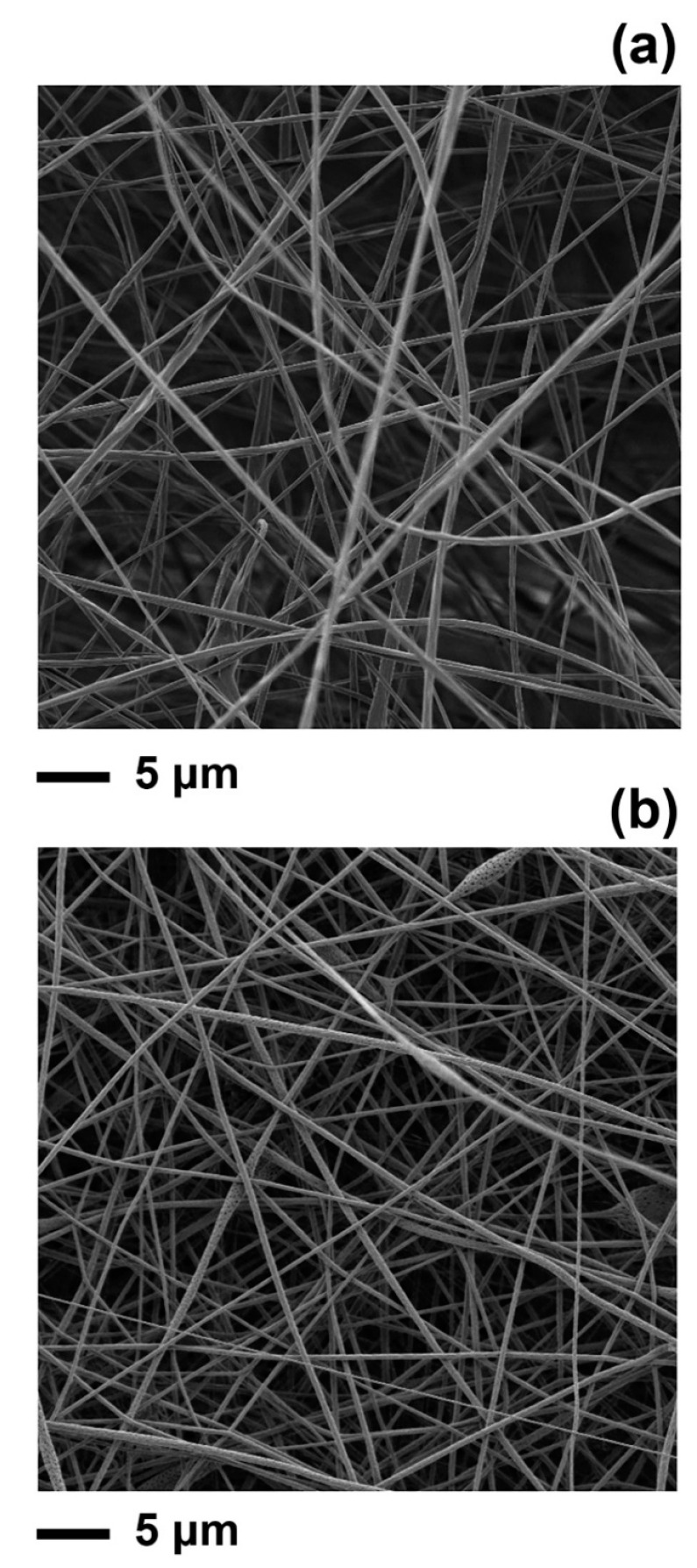
Selection of SEM images of electrospun mats of (**a**) PLA and (**b**) PLA–limonene fibres.

The PLA and PLA–limonene fibres of this work were electrospun from acetone at a polymer concentration of 14 *w*/*v*%. The PLA–acetone solution was selected because it enabled the electrospinning of fibres with a consistent, bead-free morphology (Figure 1), as shown in previous studies [3,9,10]. Limonene was mixed with the PLA–acetone solution, prior to electrospinning, at a concentration of 10 *v*/*v*%. As previously demonstrated [10], this concentration of EO can be added to PLA solutions without negatively impacting on the stability of the liquid jet during electrospinning and, therefore, on the morphology of the fibres.

### 3.1. Characterisation of PLA and PLA–Limonene Electrospun Fibres

The PLA fibres produced had an average diameter of 1002 ± 394 nm, which is in agreement with the literature [10,26], while the diameter of PLA–limonene fibres was 633 ± 304 nm. The reduction in fibre diameter measured after the addition of limonene is within the ranges seen in other studies when electrospun fibres were loaded with an EO [27].

**Figure 2:**
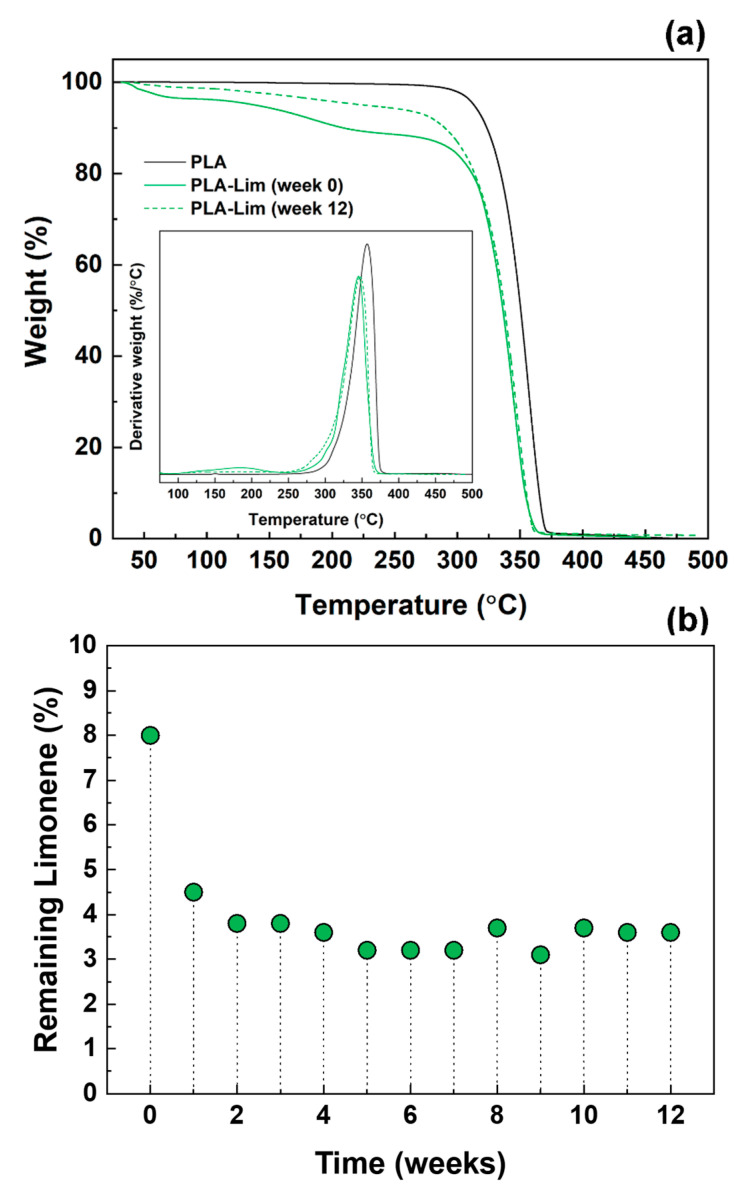
(**a**) TGA curves for electrospun mats of PLA (solid black line), PLA–limonene at time equal to 0 weeks (just after electrospinning; solid green line), PLA–limonene after 12 weeks of storage in normal conditions (dashed green line). Inset: first derivatives of the TGA curves, showing the stages of thermal degradation for the PLA–limonene fibres. (**b**) Limonene contained in the PLA–limonene fibres, at different time points, after storage in normal conditions. The remaining limonene percentage is obtained from the TGA thermograms considering the weight loss of the samples at 183 °C.

In order to investigate the long-term effect of limonene on the physical properties of the PLA electrospun fibres, the thermal stability of the fibres was analysed by TGA. This approach is commonly used to estimate the loading of EOs in polymeric systems, like chitosan nanoparticles [28,29], and poly(butylene adipate-co-terephthalate) (PBAT) capsules [30]. As shown in Figure 2a, the thermograms of the PLA fibres (without limonene) are characterised by a single thermal degradation step, with an average decomposition temperature of 356 °C that was determined from the peak of the first derivative of the TGA curve (inset of Figure 2a). This is consistent with previous studies which reported degradation temperatures of 354 °C and 366 °C for PLA electrospun fibres [31,32] and in the range of 349–370 °C for PLA films [33,34,35,36].

PLA–limonene fibres, which were analysed immediately after preparation (week 0), exhibited a first stage of weight loss below 100 °C. This can be attributed to the evaporation of the residual solvent and moisture entrapped into the electrospun mat [37,38] and possibly the traces of limonene present on the surface of the fibres. The second stage of degradation, where a high rate of weight loss was observed (peak in the first derivative curve), was at 183 °C, which is not visible in the curve of PLA fibres and corresponds to the degradation of limonene encapsulated into the electrospun fibres. Free limonene, instead, exhibited a one-step mass loss with a maximum temperature of 89 °C and it completely degraded above 100 °C, concordant with the literature [18]. The encapsulation of limonene into the electrospun fibres prevented evaporation, retaining limonene within the electrospun PLA fibres. Previous reports have noted that the encapsulation of essential oils within electrospun fibre improved their thermal stability [39,40]. For example, the degradation temperature of oregano essential oil was increased from 195 °C to 338–358 °C after encapsulation in chitosan nanoparticles [29]; similarly, the carvacrol contained in chitosan nanoparticles degraded at 340 °C, rather than at 186 °C as free carvacrol did [41]. 

The last step in the thermographs of the PLA–limonene fibres occurred at 345 °C and is linked to the decomposition of the polymeric matrix. The shift in the degradation temperature of PLA towards a lower temperature due to the addition of limonene has been reported for PLA–limonene films and is due to the interaction of the essential oil with the polymer [33,34].

### 3.2. Analysis of PLA and PLA–Limonene Electrospun Fibres over Time

As shown in Figure 2b, the percentage of weight loss from the TGA analysis was used to estimate the limonene remaining in the samples after electrospinning and storage. Immediately after preparation (week 0), 8% of limonene was recorded, and this value went down to 4.5% at week 1 and remained between 3 and 4% for the rest of the test period (from week 2 to week 12). These results indicate that the partial evaporation of limonene happened during fibre formation (possibly together with solvent removal during electrospinning), and it continued during storage at a rate that was higher within the first week and stabilised after the second week. It is expected that the limonene that was adsorbed onto the outer layers of the fibres/mats was the first to be released, while the limonene entrapped inside the inner core of the fibres was more stable and still detectable after 12 weeks. Although a direct comparison with the literature is not possible (no previous studies are available), the percentage of residual limonene detected at week 0 is in line with the values calculated (using TGA data) for Plai essential oil and PLA electrospun fibres [32], and lemon myrtle essential oil (LMEO) and cellulose acetate (CA) fibres [37]. In the first case, Plai oil content was in the range of 4.5–5.1%, starting from PLA solutions at oil concentrations of 15, 20 and 30 wt% [32]; LMEO was 5.4% and 9.5% for the mats electrospun from CA solutions with 5 and 20 wt% of LMEO, respectively [37]. The conditions and duration of storage were not considered in these studies.

**Figure 3:**
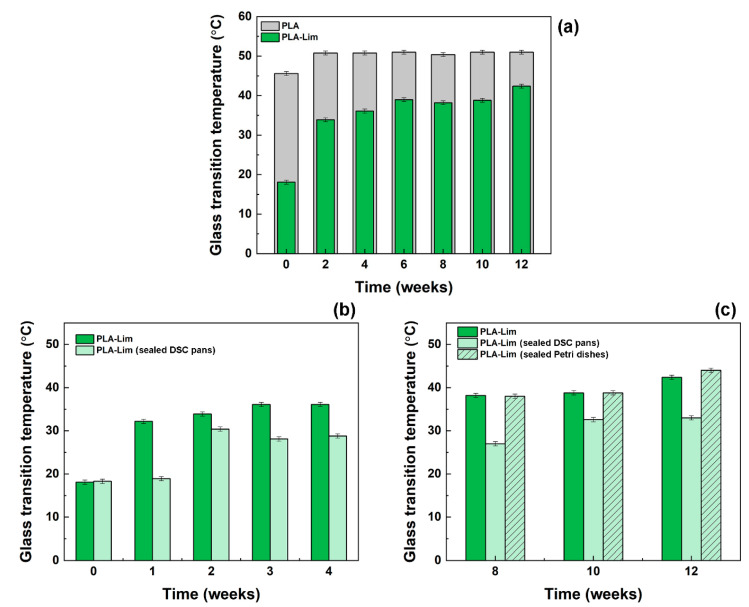
Glass transition temperature values at different time points for: (**a**) PLA fibres (grey histograms) and PLA–limonene fibres (green histograms) after storage under normal conditions (unsealed containers); (**b**) PLA–limonene fibres stored in normal conditions and PLA–limonene fibres stored in sealed DSC pans (light green histograms); (**c**) PLA–limonene fibres stored in normal conditions, PLA–limonene fibres stored in sealed DSC pans and PLA–limonene fibres stored in a sealed Petri dish (light-green, patterned histograms).

Changes in the glass transition temperature (*T_g_*) of the electrospun mats were determined by DSC measurements, over time for different storage conditions. *T_g_* values are plotted in Figure 3a for PLA fibres and PLA–limonene fibres, stored in normal conditions (unsealed containers). Representative DSC thermograms can be found in the Supplementary Material (Figures S1–S3). The *T_g_* of PLA fibres was consistent at 51 °C across the duration of the experiment, with the exception of week 0 (just after fibres’ preparation), where it was around 45 °C. This below-average value can be due to the solvent residues that were still present in the electrospun mats. In the literature, the glass transition temperature of PLA electrospun fibres (randomly oriented) is reported to be in the range of 52–56 °C, although it is not specified how and for how long the samples were stored before testing [3,42,43,44,45]. The *T_g_* of PLA–limonene fibres was 18 °C in week 0 but rose to 32 °C in just one week of storage and to 34 °C within two weeks (Figure 3b). After that, small increments were recorded in the range of 34–42 °C (from week 3 to week 12). It is expected that the evaporation of solvent and limonene, which happened primarily in the first week of storage in accordance with TGA measurements, greatly impacted on the *T_g_* change observed. The value measured in week 0 is in line with a previous work, where a glass transition temperature of 20 °C was reported for PLA–limonene electrospun fibres [9]. For all time points considered, the *T_g_* of the composite fibres was always lower than that one of PLA. This is due to the plasticising effect of limonene, which enhances the mobility of the polymer chains and alters intermolecular forces [33,34,35,46].

Different storage conditions (sealed systems instead of open ones) impacted on how the glass transition temperature of the PLA–limonene mats changed over time. When the samples were kept in sealed DSC pans immediately after preparation (Figure 3b,c), the *T_g_* remained constant just below 20 °C for weeks 0 and 1, then it increased to around 30 °C (28–33 °C) for the rest of the experiment. These values are lower than those recorded for the samples in unsealed containers, suggesting that the sealed environment limited the evaporation of the volatile components over time at room temperature. Negligible differences were instead noticed between the samples kept under normal conditions and inside sealed Petri dishes (Figure 3c), possibly because the unoccupied volume of the sealed container was larger than the one occupied by the electrospun mats (evaporation was not hindered).

**Figure 4:**
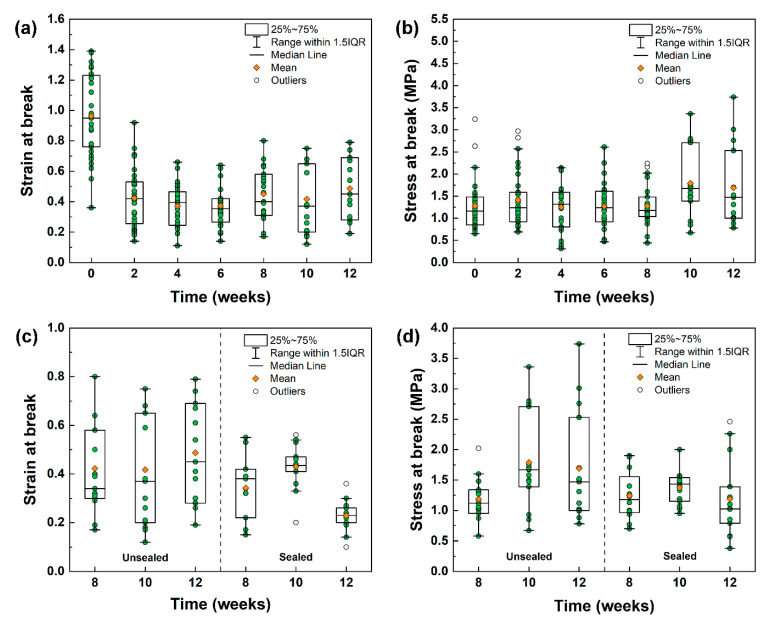
Box plots of (**a**) strain at break and (**b**) stress at break for PLA–limonene fibres stored under normal conditions for 12 weeks. Comparison between (**c**) strain at break and (**d**) stress at break for PLA–limonene fibres stored under normal conditions (unsealed, box plot on the left-hand side) and inside sealed Petri dishes (sealed, box plot on the right-hand side) for weeks 8, 10 and 12. The green symbols represent the individual measurements (repeats). Maximum and minimum values within 1.5 interquartile range (IQR) are indicated by the whiskers; lower and upper ends of the boxes represent the 25th and 75th percentile, while the middle line represents the median.

From the DSC measurements, it emerges that the glass transition temperature of the PLA–limonene mats changed greatly within the first week of storage under normal conditions and then tended to stabilise. A similar trend was observed for the mechanical properties of the composite fibres when subjected to tensile loading until failure. A number of specimens between 15 and 30 were analysed per each time point to appreciate the statistical differences between storage duration and conditions (Figure 4 and Figure S4). The samples tested just after electrospinning (week 0, no storage) were characterised by a mean strain at a break of (1.0 ± 0.3) and a mean stress at a break of (1.3 ± 0.6) MPa. After storage under normal conditions (unsealed containers), the strain at break was around 0.4–0.5 for all time points analysed. One-way analysis of variance (ANOVA) and Tukey post-hoc analysis indicated that the mean value of the strain at the break of week 0 was significantly different (significance level of 0.05) from that of weeks 2, 4, 6, 8, 10 and 12 (Figure 4a). It is expected that the electrospun samples tested immediately after production contained the highest amount of limonene (as indicated by TGA and DSC measurements), which is a natural plasticiser for PLA [33,34,35,46]. This resulted in an improved mechanical flexibility of the fibrous mats at week 0 if compared with the samples left to age, because of limonene evaporation over time. In addition, the glass transition temperature of the electrospun PLA–limonene samples at week 0 was lower than room temperature, whereas the *T_g_* values of the aged, plasticised fibres was higher than room temperature, which means that the material state at room temperature (testing conditions) changes from rubbery to glassy, with a consequent change in flexibility. No significant changes were observed instead for the stress at break (Figure 4b).

When the PLA–limonene specimens were kept in sealed containers and tested at weeks 8, 10 and 12, although their mechanical response was similar to that of the samples in unsealed containers, narrower distributions of data points were recorded for both the strain at break and the stress at break (Figure 4c,d). The reduced sample variability can be attributed to a better control over sample ageing. The data demonstrate that electrospun PLA–limonene samples were less brittle and more flexible when the limonene content was higher, which agrees with previous work analysing PLA/limonene blends [33]. 

### 3.3. Antibacterial Activity of PLA and PLA–Limonene Electrospun Fibres

**Figure 5:**
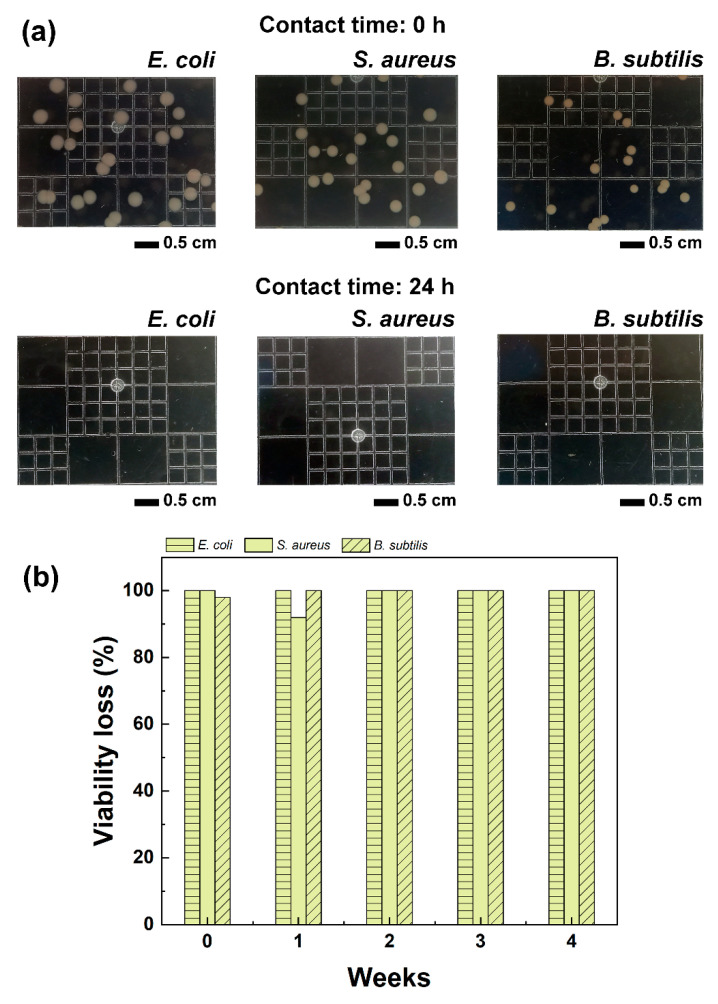
(**a**) Selected digital photographs of agar plates showing the growth of *E. coli*, *S. aureus* and *B. subtilis* after 0 h (white spots) and 24 h of incubation with PLA–limonene electrospun fibres. Similar results were obtained for all time points examined (weeks 0, 1, 2, 3 and 4). (**b**) Viability loss of *E. coli*, *S. aureus* and *B. subtilis* cultures incubated with PLA–limonene fibres that were kept in unsealed containers for up to 4 weeks.

As discussed so far, the properties (thermal and mechanical) of PLA electrospun fibres containing limonene evolved over time depending on the storage conditions and duration, primarily due to the release of limonene. Despite this, the amount of limonene retained by the fibres was sufficient to impart antibacterial activity to the electrospun mats, against the following model microorganisms: *E. coli*, *S. aureus* and *B. subtilis*. As shown in Figure 5a, when the microorganisms were exposed to the PLA–limonene fibres for 24 h, no bacteria colonies were detected (clear agar gel plates), in contrast to the PLA samples which instead had no inhibitory effect, see Figure S5. Storage time had no impact on the antibacterial activity of the samples (Figure 5b), and the PLA–limonene fibres stored for 4 weeks before testing were as effective as the freshly prepared samples (week 0). The timepoint of 4 weeks was chosen because, from TGA and DSC measurements, it emerged that the properties of the fibres stabilised after 3 weeks of storage, indicating that the limited evaporation of the entrapped limonene occurred after that period of time.

The exposure of pathogens, both Gram-positive and Gram-negative, to limonene eventually results in their death because of the disruption of the integrity and permeability of the bacteria cell wall with the consequent leakage of cellular content (proteins and nucleic acids) [47,48,49]. Also, the inactivation activity of limonene is attributed to alterations of bacterial respiration and metabolisms, and enzyme inhibition and regulation. The recent interest in limonene and citrus essential oils (which contain limonene) is linked to their inhibitory effect against a wide range of microorganisms including multi-drug resistant ones, which makes them a promising natural alternative to antibiotics [50,51,52]. 

## 4. Conclusions

Previous studies have established that essential oils can impart antibacterial, anti-inflammatory or antioxidant properties to electrospun nanofibres. In this work, the essential oil limonene was selected to be electrospun with PLA and its effect on the properties (thermal, mechanical and antibacterial) of the resulting fibrous mats was investigated over extended periods of time (up to 12 weeks) under different storage conditions (unsealed and sealed containers). It was found, by TGA measurements, that the evaporation of limonene occurred primarily during the electrospinning process and partially within the first week of storage under normal conditions where the limonene present on the surface of the fibres was mainly affected. From week 2 to week 12, the amount of limonene retained by the fibres underwent negligible changes. The inclusion of limonene resulted in changes in the thermal and mechanical properties of the PLA electrospun mats, as demonstrated by DSC and tensile testing, respectively. The glass transition temperature of the composite fibres changed with time and was correlated with the concentration of limonene. Freshly prepared PLA–limonene fibres had the lowest glass transition temperature, due to the highest amount of limonene still available to act as a plasticiser for PLA. After the first two weeks of storage (in both unsealed and sealed systems), the glass transition temperature tended to increase and stabilised after that period of time. This impacted on the response of the fibres under tensile loading, with the highest strain at break recorded for the just-produced fibres and lower values calculated for the remaining time points. Storage in a sealed environment contributed to the stability of the fibres since a more constant limonene concentration was maintained for longer. Given that it is generally advised to frequently change wound dressings to maintain a clean wound environment, typically daily to weekly, any possible degradation of the PLA–limonene fibres is expected to be low and the resulting impact on the mechanical properties negligible.

The limonene encapsulated within the PLA fibres was effective in inhibiting the proliferation of three different species of bacteria, all of which are known to cause complications in hospital settings. In this case, storage duration had a limited effect on the antibacterial properties, and the fibres were still active after four weeks of storage under normal conditions. Seeing as the amount of limonene retained within the PLA fibres from week 2 to week 12 underwent negligible changes, estimated by TGA analyses, it is thought that the antibacterial properties would be retained throughout this 12-week duration too; this suggests that the material developed here would be functional in inhibiting microbial colonisation when used in application as a wound dressing. 

Based on the results of this study, it can be concluded that the encapsulation of essential oils into electrospun fibres is an effective way to protect and deliver these bioactive compounds and generate materials with potential biomedical applications. However, the properties of the resulting electrospun fibres evolve over time due to the evaporation of the natural compounds, with a consequent impact on the thermal and mechanical stability of medical devices consisting of these composite fibres. Despite this, the degree of change seen is not expected to impact the material functionality in service, especially when considering an application like a wound dressing.

## Figures and Tables

**Table 1:** Details of the fibres produced through electrospinning, the nomenclature and the timepoints at which the fibres were tested (time since fibre generation).

Fibre Batch Details	Reference Name	Timepoints Tested (Weeks)
PLA nanofibres without any additives, stored at ~20 °C	PLA	Various between 0 and 12
PLA nanofibres loaded with 10 *v*/*v*% limonene, stored at ~20 °C	PLA–Lim	Various between 0 and 12
PLA nanofibres loaded with 10 *v*/*v*% limonene, stored in sealed conditions (in either Petri dishes or DSC pans).	Sealed PLA–Lim	Various between 0 and 12

## Data Availability

Data are contained within the article and Supplementary Materials.

## Supplementary Materials

The following supporting information can be downloaded at: https://www.mdpi.com/article/10.3390/polym16071005/s1, Figure S1: Representative differential scanning calorimetry (DSC) thermogram for blank poly(lactic acid) PLA fibres at time = 0.; Figure S2: Representative differential scanning calorimetry (DSC) thermogram for PLA-limonene fibres at time = 0, stored in open conditions.; Figure S3: Representative differential scanning calorimetry (DSC) thermogram for PLA-limonene fibres at time = 8 weeks, stored in sealed conditions.; Figure S4: Stress-strain graph comparing the failure profiles of blank PLA with PLA-Lim at timepoints 0, 4, and 8 weeks after fibre generation. Each line represents an average from 15 samples, and upper and lower bounds represent the standard error from the average.; Figure S5: Representative control results associated with the antibacterial studies performed. Showing digital photographs of *E. coli* bacterial growth after cultures are exposed to blank PLA electrospun fibres (no limonene).
