# Supplementary material for: Investigating How the Properties of Electrospun Poly(lactic acid) Fibres Loaded with the Essential Oil Limonene Evolve over Time under Different Storage Conditions
Source: Polymers (Basel). 2024 Apr 7;16(7):1005. doi: 10.3390/polym16071005 (PMC11013927; doi:10.3390/polym16071005)
Supplement: Supplementary file 1 [file polymers-16-01005-s001.zip › polymers-2880540-supplementary.pdf]

Supplementary Material

Investigating How the Properties of Electrospun Poly(lactic acid) Fibres Loaded with the Essential Oil Limonene Evolve over Time under Different Storage Conditions

Leah Williams [1,*], Fiona L. Hatton [1], Maria Cristina Righetti [2] and Elisa Mele [1,*]

1 Department of Materials, Loughborough University, Loughborough LE11 3TU, UK; f.hatton@lboro.ac.uk
2 National Research Council-Institute for Chemical and Physical Processes (CNR-IPCF), Via Moruzzi 1, 56124 Pisa, Italy; cristina.righetti@pi.ipcf.cnr.it
* Correspondence: l.williams@lboro.co.uk (L.W.); e.mele2@lboro.ac.uk (E.M.)

Supplementary materials include representative DSC thermograms for PLA and PLA-Lim fibres, representative tensile testing data (stress-strain curves) and control results associated with the antibacterial studies performed.

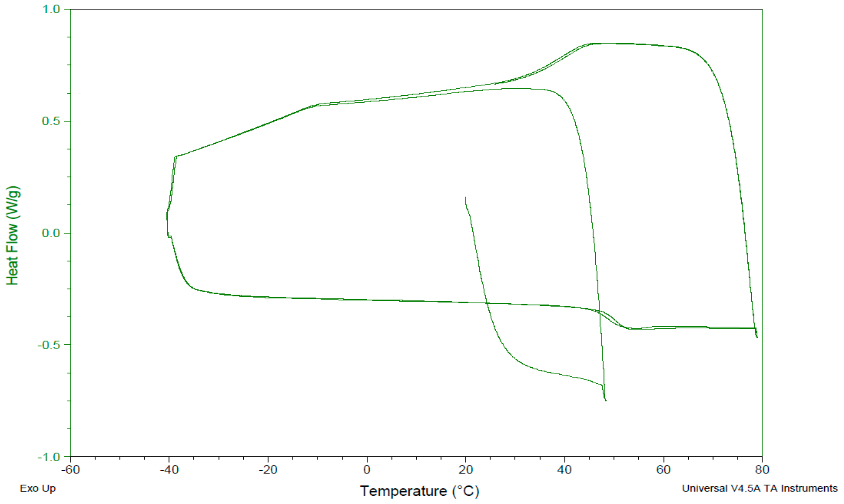

Figure S1. Representative differential scanning calorimetry (DSC) thermogram for blank poly(lactic acid) PLA fibres at time = 0.

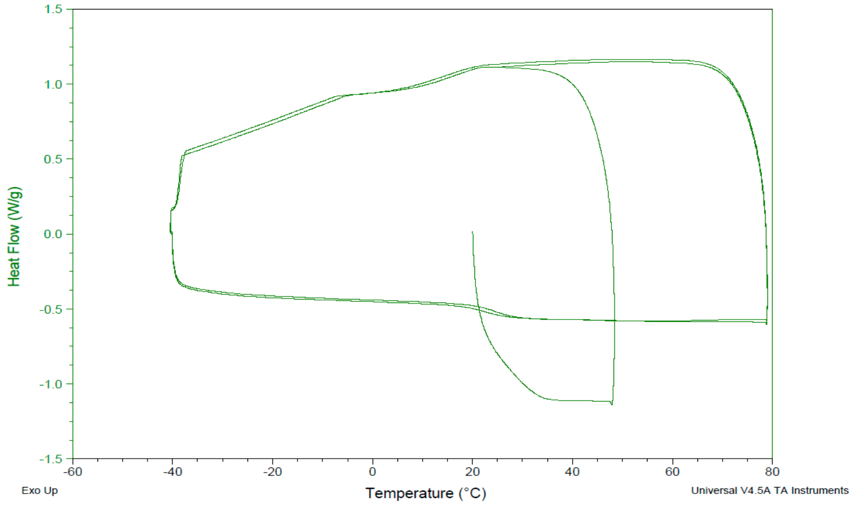

Figure S2. Representative differential scanning calorimetry (DSC) thermogram for PLA-limonene fibres at time = 0, stored in open conditions.

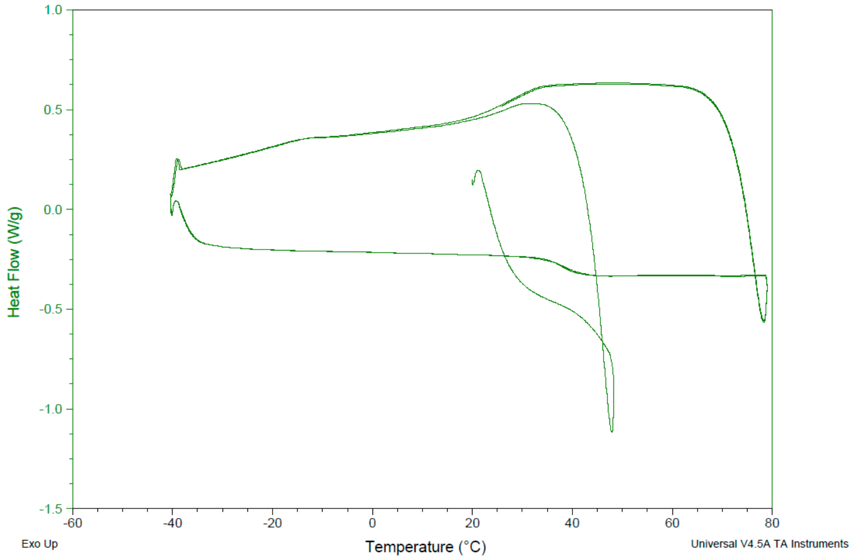

Figure S3. Representative differential scanning calorimetry (DSC) thermogram for PLA-limonene fibres at time = 8 weeks, stored in sealed conditions.

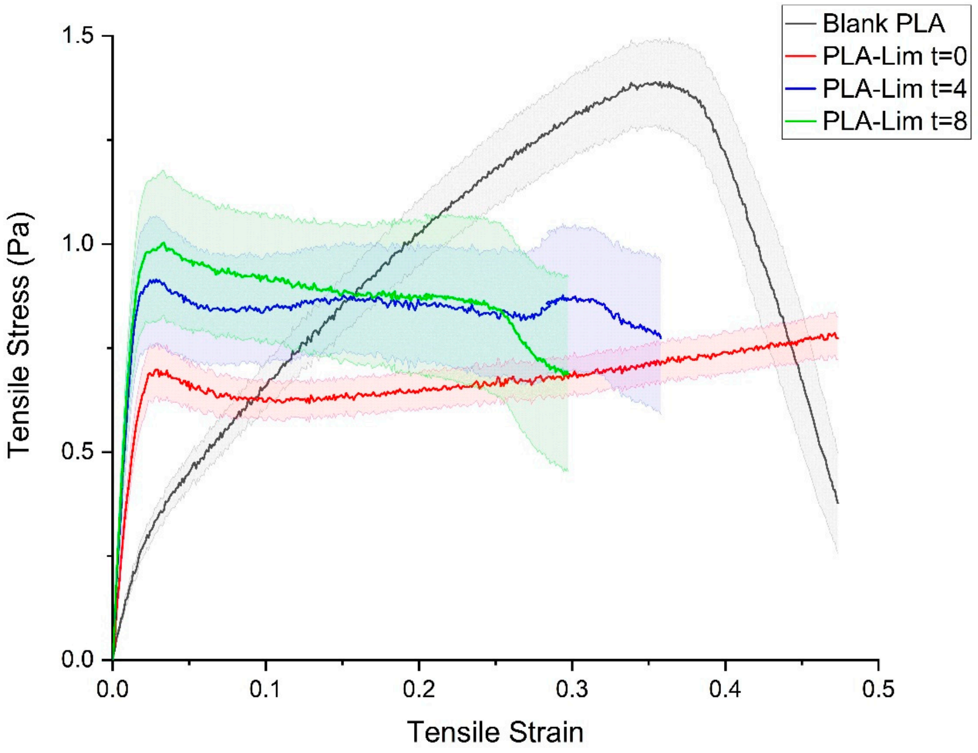

Figure S4. Stress-strain graph comparing the failure profiles of blank PLA with PLA-Lim at timepoints 0, 4, and 8 weeks after fibre generation. Each line represents an average from 15 samples, and upper and lower bounds represent the standard error from the average.

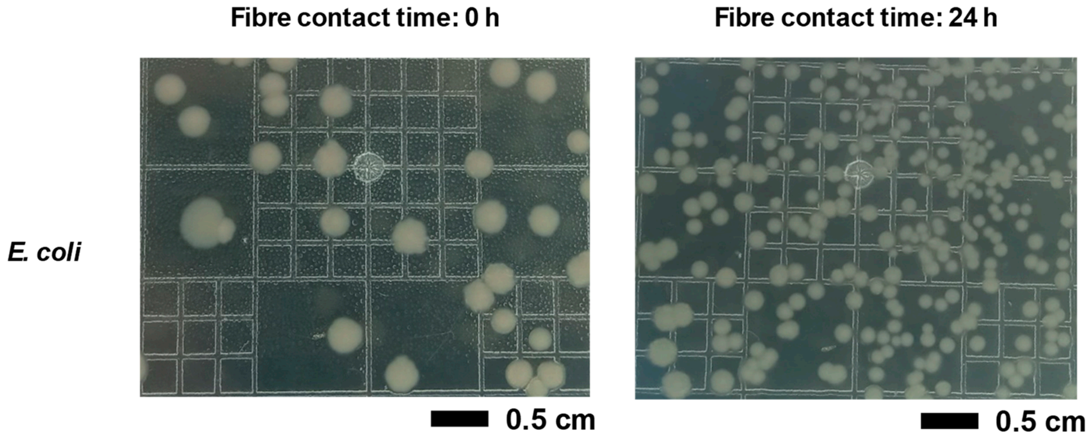

Figure S5. Representative control results associated with the antibacterial studies performed. Showing digital photographs of E. coli bacterial growth after cultures are exposed to blank PLA electrospun fibres (no limonene).
